# Amphetamine-type stimulants and HIV infection among men who have sex with men: implications on HIV research and prevention from a systematic review and meta-analysis

**DOI:** 10.7448/IAS.18.1.19273

**Published:** 2015-02-02

**Authors:** Nga Thi Thu Vu, Lisa Maher, Iryna Zablotska

**Affiliations:** 1 The Kirby Institute, The University of NSW Australia, Sydney, NSW, Australia;; 2 Centre for Social Research in Health, The University of NSW Australia, Sydney, NSW, Australia

**Keywords:** HIV, amphetamine-type stimulants, MSM, systematic review, meta-analysis, risk behaviour, meth/amphetamine, ecstasy

## Abstract

**Introduction:**

HIV infections and the use of amphetamine-type stimulants (ATS) among men who have sex with men (MSM) have been increasing internationally, but the role of ATS use as a co-factor for HIV infection remains unclear. We aimed to summarize the association between ATS use and HIV infection among MSM.

**Methods:**

We conducted a systematic search of MEDLINE, EMBASE, GLOBAL HEALTH and PsycINFO for relevant English, peer-reviewed articles of quantitative studies published between 1980 and 25 April 2013. Pooled estimates of the association – prevalence rate ratios (PRR, cross-sectional studies), odds ratio (OR, case-control studies) and hazard ratio (HR, longitudinal studies), with 95% Confidence Intervals (CI) – were calculated using random-effects models stratified by study design and ATS group (meth/amphetamines vs. ecstasy). We assessed the existence of publication bias in funnel plots and checked for sources of heterogeneity using meta-regression and subgroup analysis.

**Results:**

We identified 6710 article titles, screened 1716 abstracts and reviewed 267 full text articles. A total of 35 publications were eligible for data abstraction and meta-analysis, resulting in 56 records of ATS use. Most studies (31/35) were conducted in high-income countries. Published studies used different research designs, samples and measures of ATS use. The pooled association between meth/amphetamine use and HIV infection was statistically significant in all three designs (PRR=1.86; 95% CI: 1.57–2.17; OR=2.73; 95% CI: 2.16–3.46 and HR=3.43; 95% CI: 2.98–3.95, respectively, for cross-sectional, case-control and longitudinal studies). Ecstasy use was not associated with HIV infection in cross-sectional studies (PRR=1.15; 95% CI: 0.88–1.49; OR=3.04; 95% CI: 1.29–7.18 and HR=2.48; 95% CI: 1.42–4.35, respectively, for cross-sectional, case-control and longitudinal studies). Results in cross-sectional studies were highly heterogeneous due to issues with ATS measurement and different sampling frames.

**Conclusions:**

While meth/amphetamine use was significantly associated with HIV infection among MSM in high-income countries in all study designs, evidence of the role of ecstasy in HIV infection was lacking in cross-sectional studies. Cross-sectional study design, measurement approaches and source populations may also be important modifiers of the strength and the direction of associations. Event-specific measure of individual drug is required to establish temporal relationship between ATS use and HIV infection. HIV prevention programmes targeting MSM should consider including interventions designed to address meth/amphetamine use.

## Introduction

Amphetamine-type stimulants (ATS) are the second most popular group of illegal drugs globally and are increasingly used in different populations and in different parts of the world [1,2]. ATS can be classified into two main subgroups: meth/amphetamines, which include amphetamine sulphate, amphetamine hydrochloride, methamphetamine and methcathinone, and ecstasy subgroup, which comprises MDMA (3,4-methylenedioxy-N-methylamphetamine) and its analogue (called meth/amphetamines and ecstasy hereafter) [1,3]. Both groups are synthetic neurotropic stimulants that can be ingested orally, injected, inhaled, smoked or “shafted” (inserted in the anus) and have immediate accelerated physical and psychological effects which last up to 10–12 hours (meth/amphetamines) or 3–6 hours (ecstasy) [4,5]. Ecstasy is the most common street name for MDMA [6]. As to methamphetamine, its street slang names vary geographically, and some of them “crystal,” “speed,” “ice,” “crank,” “batu,” “glass,” “chalk” and “go-fast” [7,8].

In relation to sex, meth/amphetamine and ecstasy have been documented to have different effects. Meth/amphetamines are often used to increase sexual desire, make intercourse more pleasurable, facilitate sexual experimentation and decrease sexual inhibition [9,10]. Meth/amphetamines may increase sexual pleasure, help prolong sexual performance, facilitate sexual marathons, make anal intercourse easier and less painful, particularly during more forceful and traumatic sexual penetration [11]. Such attributes have been valued in more sexually adventurous gay community subcultures [12]. Meth/amphetamine use clearly affects both physiological and psychological aspects of sexual behaviour and may facilitate risky sexual practices, including unprotected sex, thereby increasing the risk of HIV transmission.

While some studies suggest that ecstasy use may also increase sexual satisfaction, prolong and enhance sexual arousal [13–17], other studies found no effect on sexual desire in penetrative sexual intercourse [18,19]. Ecstasy has also been reported to increase feelings of sensuality and emotional closeness [20,21]. Therefore, it may be used in the context of less risky sex and its impact on HIV transmission is less well defined.

In the past decade, ATS use has become increasingly popular among men who have sex with men (MSM) in North America, Asia, Western and South Western Europe [22–33]. In high-income countries such as the United Kingdom and the United States, the prevalence of recent (past 12 months) amphetamine use among MSM was reported to be between 7.2 and 18.8% [22,23], recent meth/amphetamine use – between 2.8 and 18.0% [23–25] and recent ecstasy use – between 18.5 and 36.7% [23,34,35]. The prevalence of lifetime use of these substances among MSM in seven US cities was found to be much higher [26,32,33]. An online study of drug use among MSM in 12 countries in Asia in 2010 reported an overall prevalence of recreational drug use over a six-month period of 16.7%, with ecstasy the most commonly used drug (8.1%) [30]. Data from studies assessing drug use during specific gay community events and venues in Western countries, (e.g. circuit parties, dance clubs, bars and bathhouses) have found the prevalence of both meth/amphetamine and ecstasy use to be even higher [34,36].

A growing body of literature documents significant associations between meth/amphetamine and ecstasy use and unprotected anal intercourse (UAI), including receptive UAI – a practice which carries the highest risk of HIV infection [11,22,28,32,37–43]. ATS use and UAI are co-occurring risk behaviours with the potential to facilitate HIV transmission among MSM. Since ATS can also be administered parenterally, exposure to HIV can also occur via unsafe injecting practices [44,45].

A number of studies have directly focused on the association between ATS use and HIV infection or included measures of ATS use in their analyses of associates/risk factors of HIV infection among MSM [8,25,42,46–77]. However, the results of these studies have been inconsistent as to the significance of this association. Furthermore, the interpretation of their findings may be complicated given the variety of study designs, sampling frames and measures of ATS use. The main objective of this systematic review and meta-analysis was to evaluate and summarize the association between ATS use and HIV infection among MSM in different study designs and by ATS subgroup (meth/amphetamines and ecstasy).

## Methods

This paper followed the guidelines for reporting a meta-analysis of observational studies (MOOSE) proposed by Stroup *et al*. [78].

### Search strategy

We conducted a systematic search in MEDLINE, EMBASE, GLOBAL HEALTH and PsysINFO for relevant publications from 1980 until 25 April 2013. The search used a combination of free terms and the Medline subject headings, including (1) MSM OR homosexual men OR bisexual men OR gay men OR male homosexual OR bisexual male OR homosexuality OR bisexuality AND (2) risk factors OR determinants OR associations OR correlates OR correlations OR predictors OR high-risk behaviours OR predictor variables AND (3) HIV prevalence OR HIV incidence OR HIV seroconversion OR HIV status OR human immunodeficiency virus OR human immunodeficiency virus prevalence/infection. Some articles reported only a combined drug use measure, did not specify the drug(s) used, did not provide a quantitative effect measure with an associated 95% confidence interval (95% CI) and did not include the original data. In these instances, we contacted the corresponding authors by email to obtain the effect measure or a descriptive tabulation of ATS use and HIV infection. If no reply was received within four weeks, the corresponding articles were excluded from further review. The search was carried out by Nga Thi Thu Vu and Julia Kennedy.

### Inclusion and exclusion criteria

Articles were eligible for inclusion in the review if they satisfied all of the following criteria: 1) cross-sectional, case-control or longitudinal study design; 2) quantitative data collection; 3) MSM as a target population; 4) the article reported a crude quantitative measure of association between ATS use and HIV infection or provided data to calculate it; 4) HIV status of participants was confirmed by a standardized laboratory method, and 5) the article was published in a peer-reviewed English language journal. Studies were excluded if: 1) they applied only qualitative methods or mathematical modelling; 2) specifically targeted only HIV positive MSM or only ATS users; 3) quantitative data could not be extracted and/or were not provided by the authors; 4) HIV status of participants was self-reported; 5) the publication included only conference proceedings; and 6) was published in a language other than English. These inclusion and exclusion criteria aimed to minimize any classification bias as to HIV status and to exclude articles which did not provide a quantitative measure of the association between ATS use and HIV infection.

### Quality assessment

The article quality was assessed using quality assessment criteria adapted for cross-sectional studies from Boyle [79] and for case-control and longitudinal studies from Wells *et al*. [80] (the checklist is provided in Supplementary 1). According to these quality criteria, a score of 1 was assigned for each of the items included and articles were assigned a summative score on a scale of 0 to 9 for cross-sectional studies, 0–10 for case-control studies and 0–11 for longitudinal studies. All scores were categorized into high- and low-quality groups based on the cut off of 50%.

### Data extraction

Extracted information included the primary author, year of publication, country of research, sampling method(s), sample size, type of drug(s) examined and recall periods, basic participant characteristics (e.g. age, sexual identification) and either a crude measure of association with 95% CI or data to calculate it. If articles reported more than one drug or used more than one recall period, each measure of drug use at each recall period was extracted as a separate record. Measures of association reported without 95% CIs were not extracted. Extracted data from cross-sectional and case-control studies were used to calculate prevalence rate ratios (PRR) [81] and odds ratios (OR), respectively. For longitudinal studies, we directly extracted hazard ratios (HR) or relative risk (RR) with 95% CI as a measure of association between ATS use and HIV seroconversion. Data extraction was carried out by Nga Thi Thu Vu and Julia Kennedy.

### Statistical analysis

Meta-analysis was performed using STATA 13.0 (StataCorp, College Station, TX, USA) and was stratified by study design and ATS subgroup (meth/amphetamine vs. ecstasy). We did not combine effect measures (i.e. PRR, OR and HR) of all selected studies because of differences in the nature and calculation methods for each of these measures. In the group of longitudinal studies, all articles reported HR as a measure of association, and only Burcham *et al*. [74] used RR. We treated this RR as equivalent to HR. The pooled estimates of the association and their 95% CI were estimated using random-effects models, as suggested by DerSimonian and Laird. Heterogeneity was defined by Q statistic when *p*>0.1 as Hardy *et al*. [82] had previously reported this method to have low power. Based on the I^2^ classification suggested by Higgins and Thompson [83], we used the cut-offs of 25, 50 and 75% to define low, medium and high levels of heterogeneity, respectively [84]. Sources of heterogeneity were checked using subgroup analysis and meta-regression [85]. The variables for meta-regression included the study quality score (high vs. low), ATS group (meth/amphetamines vs. ecstasy) and study location (high vs. low- and middle-income countries (LMIC), as according to World Bank income classification, sampling location (clinic based vs. other), drug use recall period (recent use vs. lifetime use), injecting drug use reporting (Yes vs. No) and other specific drug use measurements, that is, nitrite inhalants, heroin, cocaine and EDM use (Yes vs. No)****. Injecting drug use, specifically needle and syringe sharing and these specific drug use behaviours were assessed because they were found to be associated with HIV infection and/ or unprotected risky sexual behaviours [45,69,72,86,87], therefore, may be confounders of the association between ATS use and sexually transmitted HIV infection. Sensitivity analysis was performed by the Comprehensive Meta-Analysis software V2.0 (Biostat, Englewood, New Jersey) to explore any possible influence of abnormal or outlier data on pooled estimates. Publication bias and the effects of small sample sizes were evaluated in a funnel plot [88]. Asymmetry of the funnel plot was tested as recommended by Egger *et al*. [89].

## Results

**Figure 1:**
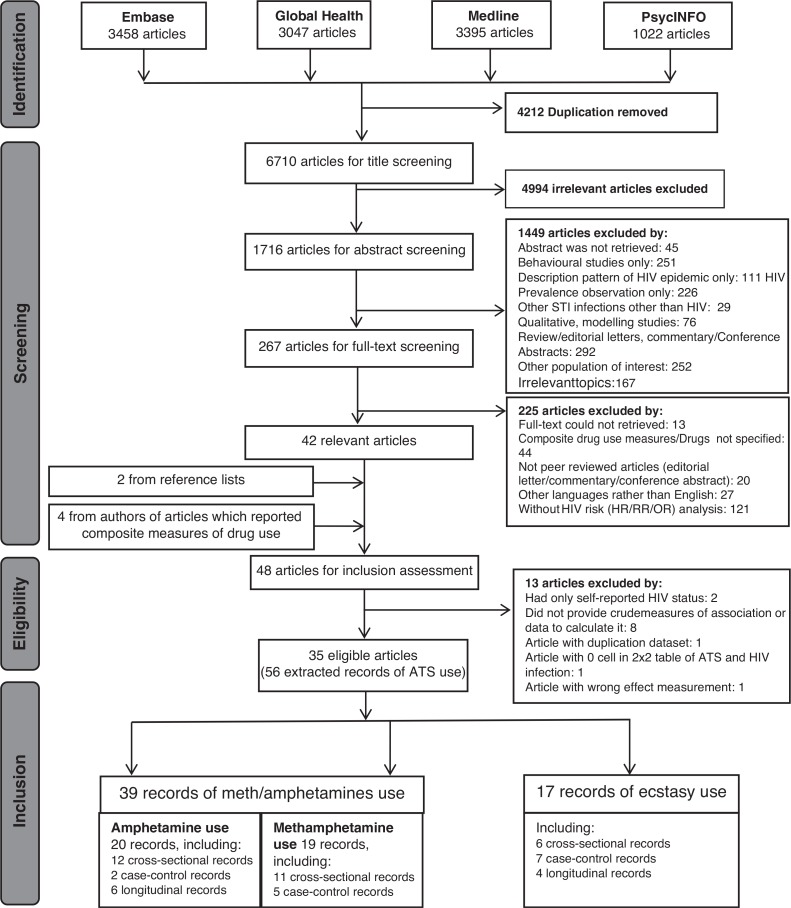
Flow chart for selection of studies with number of articles.

The flow of the review process is shown in Figure 1. We identified 6710 unique article titles, 262 of which progressed to full text screening, resulting in 42 articles relevant for this review (list of excluded articles is provided upon request). The review yielded six additional articles: two from reference lists [69,77] and four from corresponding authors of articles reporting only composite measures of drug use [46,47,53,59]. We contacted by email 30 authors of manuscripts which reported composite drug use measures and received seven responses: four [46,47,53,59] responded with tabulations of ATS use and HIV infection and three clarified that ATS had not been measured in their study or was not analyzed [90–92]. Seven articles provided a descriptive tabulation of ATS and HIV without analysis of the association with HIV infection [25,42,49,54,64,65,72]. Because some articles reported more than one drug used and/or more than one recall period, 58 records were extracted from 36 articles [8,25,42,46–77]. Two records were excluded from analysis because of a 0 cell for a 2x2 table [49,93]. Finally, 56 records from 35 studies were retained for meta-analysis. Records from Van Griensven *et al*. [46], Menza *et al*. [54] and Chesney *et al*. [69] were taken from the baseline data of their longitudinal studies; therefore, these records were treated as a cross-sectional design such that PRR was calculated for these records. The HR reported in Chesney [69] were not comparable with that measurement in other studies; therefore, these HR were not included in the analysis.

### Description of the selected studies and their participants

Among 35 selected articles, only five were from low- and middle-income countries (LMIC), while 30 were from the United States and other high-income countries, specifically The Netherlands, Australia and the United Kingdom. The majority of studies (30/35) used convenience, non-random sampling, and recruited participants using such approaches as advertising, community outreach, referrals from gay community and networks, clients of MSM-specific clinics or HIV testing centres. Nine studies used purely clinic-based recruitment, sixteen used community-based recruitment and ten used both. Most of the articles (26/35) reported a global measure of drug use with different recall periods, including 1, 3, 6, or 12 months and lifetime use; five articles [8,51,55,58,63] reported a contextual measure of ATS use in relation to sex, and the remaining articles did not specify the recall period. Almost all of the study had a quality score larger than 50%, only seven studies, among which one from the LMIC countries, had a quality score lower than 50%.

Table 1 presents the characteristics of studies selected for meta-analysis and their participants. Regarding ATS use, the majority of articles reported the use of methamphetamine (n=19), amphetamine (n=14), ecstasy (n=14) and speed (n=3). Almost all articles (n=34) also reported the use of other drugs of which the most popular reported drugs including cocaine (n=24), nitrites/poppers (n=23), marijuana (n=18) and alcohol (n=17) and heroin (n=13). Among 29 cross-sectional studies, 25 reported high HIV prevalence (9–34%) and only five reported HIV prevalence of less than 9%. All longitudinal studies found an HIV incidence between 1.90 and 2.55 per 100 person years.

**Table 1:** Articles in the analysis (n = 35): description of studies and their participants

Author, year^a^	Country^b^	World Bank ranking^b^	Data collection period	Study type^c^	Quality score	Sampling (method, sample size)	Age mean (SD)/median (range)	Sexual orientation (%)	Reporting IDU (%)	Drug use measure		
	
										Recall period	ATS use (%)	Other drug use
Van Griensven *et al*. 2013 [46]	Thailand	1	2006	1	77.8	CS: 1744	Baseline: Median: 26 (18–56)	NR	NR	Sexual: P4M drug: lifetime; P4M	Lifetime: Ecs: 7.4 Meth: 11.2 P4M: Ecs: 3.3 Meth: 6.0	Alcohol; nitrite; EDM5
Pham *et al*. 2012 [47]	Vietnam	1	8–12/2009	1	55.6	CS: 381	Median: 20.4 (18–25.1)	Gay: 39.6; trans: 20.0; Hetero: 40.4	Yes (16.5%)	Sexual: P1M drug use: lifetime	Meth: 16.7	IDU alcohol
Ackers *et al*. 2012 [48]	USA	2	6/1998–10/1999	3	72.7	CS: 4684	Baseline: median: 35 (18–62) 18–30: 25.0%	NR	Yes (baseline: 0.23%)	P6M	Baseline: Amp: 9.0	IDU; crack; cocaine; poppers; tranquilizers; EDM5; hallucinogens; alcohol
Oster *et al*. 2011 [49]	USA	2	2–4/2008	2	50.0	CS: 110	Mean: 21	Case/control: gay: 76.0/61.0; bisexual: 12.0/27.0; hetero & other: 12.0/12.0	Yes (0.0%)	P12M	Case/control: Ecs: 4.0/79.0 Meth: 0.0/7.0	IDU; other non-injection drugs
Chariyalertsak *et al*. 2011 [50]	Thailand	1	2008–2009	1	44.4	CS: 551	<30: 88.7%	Gay: 56.1 bisexual: 18.5 trans: 25.4	NR	Lifetime	Meth: 12.7	Marijuana; heroin
Morineau *et al*. 2011 [51]	Indonesia	1	8–11/2007	1	55.6	TLS, RDS: 749	NR	NR	NR	1–3 months	Meth: 14.6	NR
Truong *et al*. 2011 [52]	USA	2	1/2004–12/2006	1	77.8	CS: 6859	NR	NR	Yes (NR)	P12M	Amp (NR^a^)	IDU
Forrest *et al*. 2010 [25]	USA	2	2004–2005	1		TLS: 946	NR	NR	Yes (3.0%)	P12M	Meth: 18.0 Ecs: 17.8	Viagra; IDU
Feng *et al*. 2010 [53]	China	1	3–7/2007	1	66.7	CS: 513	Median: 24 (16.8–44.5)	Gay: 72.9; bisexual: 25.34; hetero: 7.02	NR	Sexual: P6M drug: NR	Amp: 13.3	IDU; ketamine; alcohol; heroin
Menza *et al*. 2009 [54]	USA	2	10/2001–5/2008	1	77.8	CS: 1903	<40: 79.72%	NR	NR	P6M	Meth: 6.73	Nitrite; crack/cocaine
Carey *et al*. 2009 [55]	USA	2	2003–2005	2	60.0	CS: 444	≤30: 47.5	NR	No	Sexual:P6M drug use: sex-related drug use/P6M	Case/control: Meth: 28.8/11.4 Ecs: 6.3/4.5	Alcohol; ketamine; GHB; viagra; poppers; marijuana; cocaine; LSD; heroin
Drumright *et al*. 2009 [56]	USA	2	5/2002–2/2006	2	50.0	CS: 145	Median: 32	NR	No	Sexual: P12M drug: P12M; sex-related drug use; P12M	Case/control: P6M: Meth: 28.8/11.4 Ecs: 6.3/4.5 Sex-related/3 partners: Meth: 44.2/28.2 Ecs: 14.0/8.5	Nitrite; marijuana; GHB; Cocaine; EDM5
Rudy *et al*. 2009 [57]	USA	2	2006–2007	1	44.4	CS: 6435	18–24: 15.0% 25–34: 37.0% ≥35: 48.0%	NR	NR	Sexual: P3M drug: P12M	Meth: 13.0	EDM; nitrite; Ecs; ketamine
Thiede *et al*. 2009 [58]	USA	2	7/2002–5/2005	2	60.0	CS: 142	<30: case: 31.3% control: 40.0%	Gay: case: 96.6 control: 76.4	Yes (10.6%)	P6M	Case/control: Meth: 43.4/12.7 Ecs: 18.8 /0.9	IDU; popper; viagra; ketamine; GHB; cocaine; alcohol
Prestage *et al*. 2009 [59]	Australia	2	6/2001–12/2004	3		CS: 1427	Baseline: 37 (18–75)	Homosexual: 95%	No	P6M	Meth: 38.4; Ecs & other ATS: 58.9	Cocaine; cannabis; heroin; EDM; barbiturates; amyl nitrite; psychedelics
Raymond *et al*. 2008 [60]	USA	2	10/2003–12/2004	1	88.9	TLS: 794	18–30: 41%	Gay: 83.0; Bisexual: 15.0; Hetero: 1.0; Other: 1.0	NR	Sexual: P6M Drug: P12M	Ecs: 6.9 Speed: 14.1	Cocaine; Marijuana; Crack; Poppers
Macdonald *et al*. 2008 [61]	UK	2	9/2002–10/2004	2	70.0	CS: 232	Mean: Case: 35.2 (20–58) Control: 35.1 (20–66)	Gay: 77.0	Yes (Case: 8.0%, Control: 3.0%)	P2Y	Case/Control: Meth: 16.0/13.0, Ecs: 67.0/44.0, speed: 25.0/18.0	Alcohol; Nitrite; Cocaine; Cannabis; Ketamine; Viagra; GHB; LSD; Valium
Schwarcz *et al*. 2007 [42]	USA	2	6/2002–1/2003	1		RS: 1976	Median: 42 (18–92)	NR	NR	NR	Meth: 16.8	Viagra; Nitrite; Nocaine; other club drugs (Ketamine, Ecstasy, GHB)
Plankey *et al*. 2007 [62]	USA	2	4/1984–9/1991 & 10/1996–9/2004	3	63.6	CS: 4003	Baseline: Mean: 34.4 (SD: 8.6)	NR	Yes (baseline: 17.0%)	P6M	Baseline: Meth: 23.0 Ecs: 12.0	Poppers; Cocaine
Koblin *et al*. 2006 [63]	USA	2	1/1999–2/2001	3	63.6	CS: 4295	Baseline: Mean: 34 ≤25: 19.0%	NR	Yes (baseline: 10.0%)	P6M	Baseline: Amp: 12.3	Alcohol; IDU; non-injection drugs
Fuller *et al*. 2005 [64]	USA	2	8/2000–2/2004	1	55.6	CS: 95	Median: 28 (18–40)	Gay/bisexual: 72.0; Hetero: 28.0	Yes (25.0%)	Sexual: P2M Drug: life-time	Meth: 9.0 Ecs: 20.0	IDU; heroin; cocaine; crack
Kral *et al*. 2005 [65]	USA	2	1998–2002	1	77.8	TS: 357	<30: 22.0%	Gay: 34.0; bisexual: 44.0; hetero: 22.0	Yes (sharing needle: 84.0%)	P6M	Amp: 79.0	IDU; heroin; cocaine; crack
Buchbinder *et al*. 2005 [66]	USA	2	4/1995–5/1997	3	63.6	CS: 3257	Enrolment: ≤35: 34.6%	NR	Yes (baseline: 1.5%)	P6M	% visit: Amp: 8.8	Nitrite; cocaine; hallucinogens; IDU
Robertson *et al*. 2004 [67]	USA	2	4/1996–12/1997	1	66.7	RS: 475	<30: 65.0%	Gay/bisexual: 75.5; Hetero: 24.5	Yes (58.3%)	Life-time	Meth: 46.4	IDU, heroine, cocaine
Weber *et al*. 2003 [68]	Canada	2	1995–12/2000	3	45.5	CS: 673	Baseline: median: 25 (22–28)	NR	Yes (NR)	P11M	Meth (NR); Ecs (NR)	Crack; cocaine; poppers; marijuana; alcohol
Chesney *et al*. 1998 [69]	USA	2	1985	1	70.0	CS: 337	Mean 34.8–36	NR	NR	P6M	Amp: 19.3	Alcohol; marijuana; nitrite; cocaine; barbiturate; hallucinogens; heroin
Molitor *et al*. 1998 [68]	USA	2	7/1994–12/1995	1	66.7	CS: 32,321	Mean 28	Gay: 49.6; bisexual: 50.4	NR	Sex-related drug use	Meth: 3.5	NR
Ruiz *et al*. 1998 [70]	USA	2	2–11/1994	1	66.7	CS: 824	17–22: 50.6% 22–25: 49.4%	NR	Yes (sharing needle: 6.4%)	P6M	Ecs: 22.6 Amp: 44.1	Poppers; crack; cocaine; heroin; IDU
Page-Shafer *et al*. 1997 [71]	USA, Australia, Canada, Holland	2	1982–1985	2	60.0	CS: 690	Mean: 35.3 (7.7)	NR	No	P6M	Case/control: Amp: 26.9/13.3	Cannabis; nitrite; alcohol
Buchbinder *et al*. 1996 [72]	USA	2	1/1993–7/1994	3	77.8	CS: 1975	Baseline: median: 31	NR	Yes (NR)	P6M	Baseline: Amp/P12M: 15.7	IDU; cocaine; popper; marijuana; barbiturate
Seage *et al*. 1992 [73]	USA	2	5/1985–12/1988	1	66.7	CS: 481	<30: 34.1	NR	NR	P5Y	Amp: 28.5	Marijuana; nitrite; cocaine; heroin; LSD; PCP; barbiturate; methaquolone; nitrous oxide
Burcham *et al*. 1989 [74]	Australia	2	1/1984–7/1987	3	45.5	CS: 643	Enrolment: HIV seroconverts: Mean 33 (17–65) HIV negative: 34 (15–64)	NR	No	P6M	Amp (NR) Ecs (NR)	Cocaine; nitrite; marijuana
Rietmeijer *et al*. 1989 [75]	USA	2	11/1982–12/1985	1	55.6	CS: 216	<30: 40%	NR	Yes (17.8%)	Not specified	Amp: 66.4	IDU; alcohol; marijuana; nitrites; cocaine; LSD; heroin; barbiturate; alcohol
Van Griensven *et al*. 1987 [76]	Holland	2	10/1984–05/1985	1	33.3	CS: 741	Mean: 35	Bisexual: 34.0; gay 34	NR	Not specified	Amp: 3.0	Marijuana; nitrite; cocaine; LSD
Jeffries *et al*. 1985 [77]	Canada	2	11/1982–2/1984	2	50.0	CS: 448	Mean: 32	NR	No	P8M	Case/control: Ecs: 65.0/44.0	LSD; cocaine; marijuana; nitrite

a Number in the reference list.

b World Bank's country name (USA: United States; UK: United Kingdom); World Bank ranking, 1: low- and middle-income country, 2: high-income country.

c 1: cross-sectional study; 2: case-control study; 3: longitudinal study.

NR: not reported; CS: convenience sampling; TS: targeted sampling; RS: random sampling; TLS: time location sampling; RDS: respondent driven sampling; IDU: injecting drug users; Trans: transgender; hetero: heterosexual; P1M: past one month; P2M: past two months; P3M: past three months; P4M: past four months; P6M: past six months; P8M: past eight months; P11M: past 11 months; P12M: past 12 months; P2Y: past two years; P5Y: past five years; Meth: methamphetamine; Amp: amphetamine; Ecs: ecstasy; ATS: amphetamine-type stimulants; EDM: erectile dysfunction medications; GHB: gamma hydroxybutyrate; LSD: lysergic acid diethylamide; PCP: phencyclidine.

Seventeen of 35 articles reported injecting drug use (eight of 21 cross-sectional, three of seven case-control and six of seven longitudinal studies) and just three measured needle and syringe sharing. Prevalence of injecting drug use varied markedly between 0 and 58%. Out of eight articles which investigated the relationship between injecting drug use and HIV infection, seven found a significant univariate association. Only three articles confirmed a significant association between ATS use and HIV infection when injecting drug use was included in the model.

### Association between ATS use and HIV

**Figure 2:**
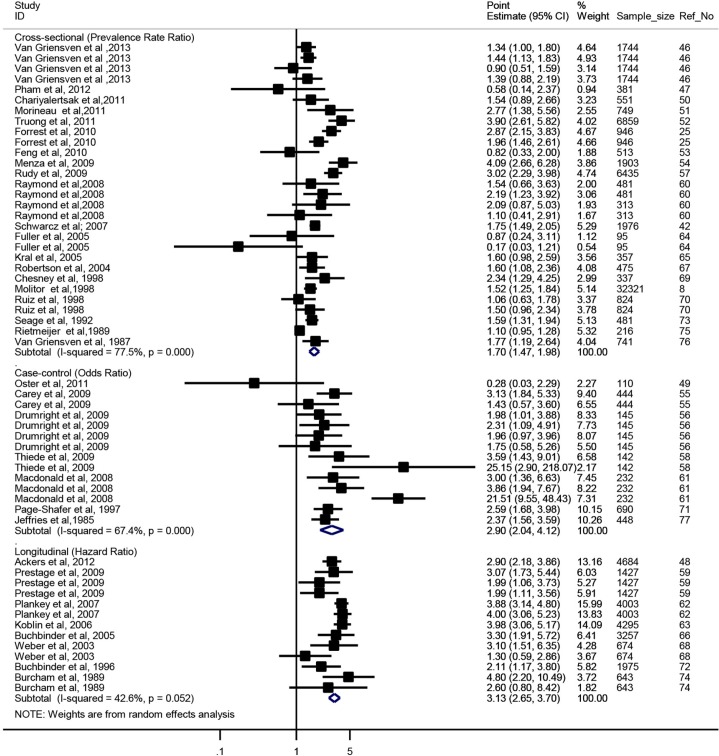
Summarized effect measure of the association between ATS use and HIV infection, by study design.

Association between ATS and HIV infection was significant *in* all study designs (Figure 2). In cross-sectional studies, MSM who reported ever using ATS were 1.70 times more likely to be infected with HIV than non-users (PRR=1.70; 95% CI: 1.47–1.98). Results in cross-sectional studies were highly heterogeneous (Q_28_=124.68, *p*=0.000 and I^2^=77.5%). In case-control studies, the pooled OR was 2.90 (95% CI: 2.04–4.12), with high heterogeneity (Q_13_=39.89, *p*=0.000 and I^2^=67.4%). In longitudinal studies, the pooled HR was 3.13 (95% CI: 2.65–3.70) with medium heterogeneity (Q_12_=20.92, *p*=0.052 and I^2^=42.6%).

**Figure 3:**
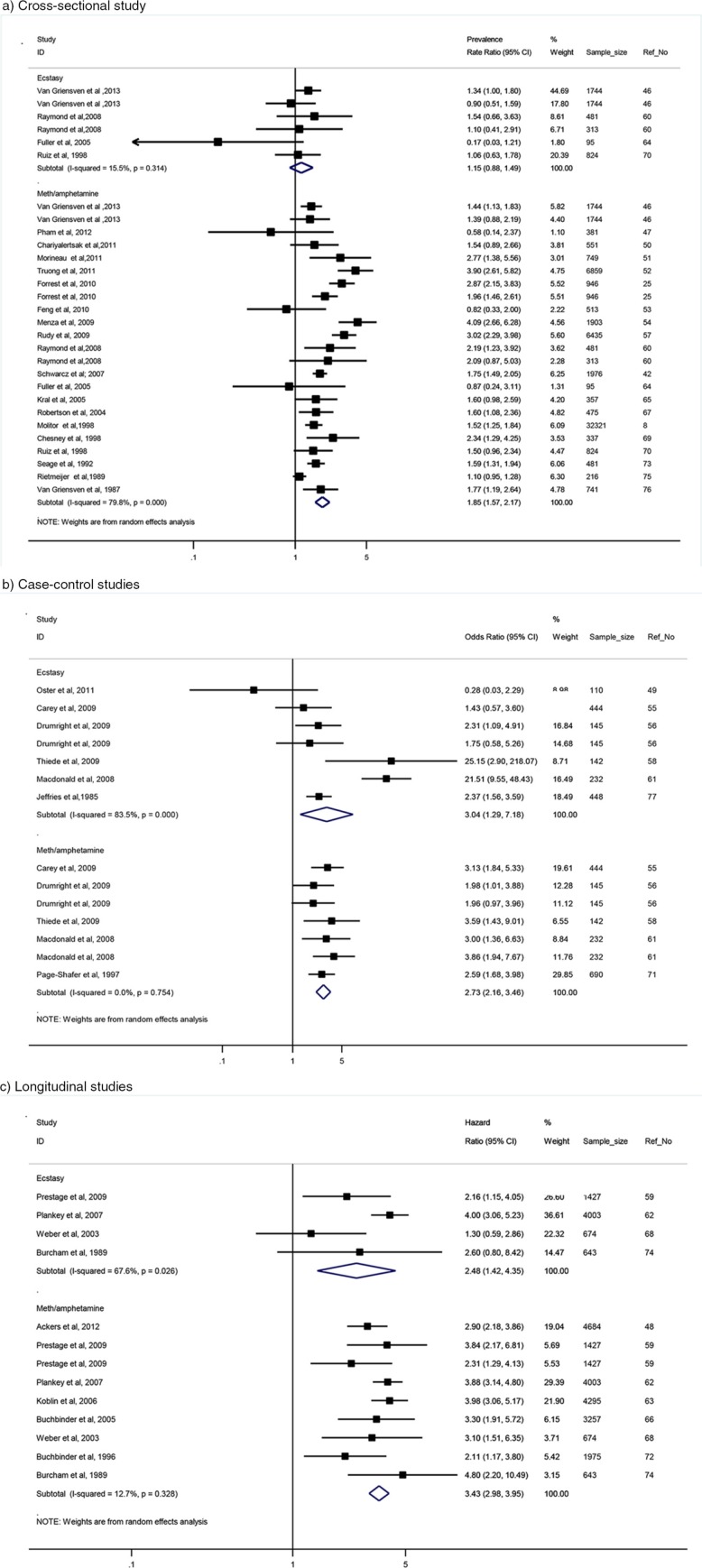
Summarized effect measure of the association between ATS use and HIV infection, by study design and drug type. (a) Cross-sectional study; (b) case-control studies; (c) longitudinal studies.

In the meth/amphetamine subgroup (Figure 3), the pooled estimate was statistically significant in all study designs (PRR for cross-sectional studies was 1.85; 95% CI: 1.57–2.17; OR for case-control studies was 2.73; 95% CI: 2.16–3.46 and HR for longitudinal studies was 3.43; 95% CI: 2.98–3.95). Heterogeneity in longitudinal and case-control studies was low (Q_7_=9.17, *p*=0.328, I^2^=12.7% and Q_5_=3.42, *p*=0.754, I^2^=0.0%, respectively) while the results of cross-sectional studies were highly heterogeneous (Q_22_=109.11, *p*<0.001 and I^2^=79.8%). However, in the ecstasy subgroup (Figure 3), in cross-sectional studies, the pooled PR estimate was not statistically significant (PR=1.15; 95% CI: 0.88–1.49), with low heterogeneity (Q_5_=5.92, *p*=0.314 and I^2^=15.5%). In case-control studies, the pooled OR estimate was significant (OR=3.04 (95% CI: 1.29–7.18), with *high* heterogeneity (Q_5_=36.33, *p*=0.000 and I^2^=83.5%). Similarly, the pooled HR estimate was statistically significant (HR=2.48; 95% CI: 1.42–4.35), with high heterogeneity (Q_3_=9.26, *p*=0.026 and I^2^=67.6%). Sources of heterogeneity among cross-sectional studies were presented in Table 2. Due to the limited number of selected case-control and longitudinal articles and because of low power of Q statistic [82], the test of heterogeneity in these study designs was not conducted.

**Table 2:** Stratification analysis for cross-sectional studies

Study characteristic	No. of records	Meta-regression (β, *p-*value)^a^	Pooled PR (95% CI)^b^
Study location			
LMIC countries	8	β=0.72, *p*=0.086	1.36 (1.12–1.65)
High-income countries	21		1.85 (1.54–2.21)
Study quality			
Low	3	β=1.34, *p*=0.289	2.10 (1.36–3.27)
High	26		1.66 (1.42–1.94)
Sampling locations			
Clinic-based sample	6	β=1.66, *p*=0.005	2.53 (1.70–3.77)
Other venues	23		1.52 (1.32–1.76)
Drug use recall period			
Recent use	19	β=1.42, *p*=0.047	1.93 (1.57–2.37)
Lifetime use	9		1.38 (1.19–1.61)
Reported injecting drugs			
No	20	β=1.02, *p*=0.914	1.70 (1.46–1.97)
Yes	9		1.62 (1.09–2.42)
Type of ATS			
Amphetamines	23	β=0.59, *p*=0.02	1.85 (1.57–2.17)
Ecstasy	6		1.15 (0.88–1.49)
Reported alcohol use			
No	21	β=0.69, *p*=0.039	1.90 (1.61–2.45)
Yes	8		1.30 (1.10–1.54)
Cocaine use			
No	13	β=0.74, *p*=0.106	1.78 (1.40–2.25)
Yes	16		1.63 (1.34–1.98)
Heroin use			
No	17	β=1.04, *p*=0.84	1.96 (1.63–2.36)
Yes	12		1.35 (1.12–1.64)
EDM use			
No	21	β=0.60, *p*=0.003	1.67 (1.37–2.03)
Yes	8		1.77 (1.40–2.24)

a Significant *p-value* indicates significant source of heterogeneity. Results from meta-regression analysis.

b Results from subgroup analysis.

LMIC: low- and middle-income countries.

### Sources of heterogeneity in cross-sectional studies

The results of subgroup analysis are presented in Table 2. Sampling locations, ATS subgroup, recall period for drug use, reporting EDM use and alcohol consumption were responsible for a high heterogeneity of the results in cross-sectional studies. The pooled estimates of the association between ATS use and HIV were significantly higher in studies which recruited participants in clinics rather than in other locations; used measures of recent versus lifetime drug use; reported EDM use (yes vs. no) or alcohol consumption (yes vs. no). Finally, the pooled PRR is higher in studies that reported meth/amphetamine use versus ecstasy use.

### Sensitivity analysis

None of the individual study results noticeably affected the pooled estimate for longitudinal and cross-sectional studies. In relation to case-control studies, the pooled OR decreased by 13.6% (from OR=2.9; 95% CI: 2.04–4.11 to OR=2.51; 95% CI: 2.02–3.12) when one high OR of a record of ecstasy use reported by Macdonald *et al*. [61] was excluded from the analysis. This record explained 50.4% of the heterogeneity of the results.

When restricted to the ecstasy subgroup among case-control studies, the pooled estimate of the association with HIV infection was also noticeably affected by the same record, which was responsible for 34.9% of the heterogeneity. After excluding this record, the pooled OR decreased by 32.2% (from OR=3.04; 95% CI: 1.29–7.18 to OR=2.06; 95% CI: 1.19–3.58).

### Publication bias

The funnel plot of all selected studies (Supplementary 2) indicates potential publication bias. However, the result of the test for symmetry of the funnel plot was not statistically significant, suggesting no small sample size effect.

## Discussion

Our review and meta-analysis of the published evidence found a statistically significant relationship between ATS use and HIV infection. The use of meth/amphetamines was significantly associated with HIV infection in all study designs, while ecstasy use was not associated with HIV in cross-sectional studies. The pooled estimate from case-control studies had low heterogeneity and the significant pooled HR from longitudinal studies was affected by studies with large samples and highly significant results [62]. The pooled estimates of case-control studies were affected by a record from study of Macdonald *et al*. [61]; however, while the exclusion of this record in the analysis resulted in decreasing the effect size; it did not change the significance of the overall effect size. The pooled estimates of cross-sectional studies were heterogeneous as a result of sampling location approach, different drug use recall periods and the diversity of different drug use measurement. Our findings of the relationship between ATS and HIV infection are consistent with results from a previous review by Drumright *et al*. [45]; that review covered fewer studies. It found that meth/amphetamine use was associated with HIV infection and reported insufficient evidence of an association between ecstasy and HIV infection. More recently, a meta-analysis of the relationship between ecstasy use and risky sexual behaviour by Hittner *et al*. found ecstasy use to be significantly associated with behaviours associated with HIV infection [20], but that review combined different sexual outcomes and did not specifically focus on MSM. Our finding of consistently significant pooled estimates of the association between meth/amphetamine use and HIV infection in all study designs proves the robustness of this association and echoes the finding of Vosburgh *et al*. [87] that methamphetamine was associated with event-level measurement of sexual risk behaviour among MSM.

Differences in the relationship between meth/amphetamine and ecstasy with HIV infection can potentially be explained by their different sexual behavioural effects. Previous research has found that meth/amphetamines facilitate sexual disinhibition and experimentation [9], increase sexual desire and facilitate sexual marathons [11] in which men practice prolonged sexual encounters with different sexual partners for hours and days [94]. Prestage *et al*. found that meth/amphetamines have often been combined with orally administered erectile dysfunction medications to further enhance sexual performance [59]. Unprotected sex is common in these contexts, as are lesions due to forceful sexual penetration and increased likelihood of condom failure, all of which can increase the risk of sexual transmission of HIV [94]. Furthermore, high dose of methamphetamine was found to increase anal sensation for receptive partners, thus promoting receptive positioning in anal sex which is the practice of highest risk in sexual transmission of HIV among MSM [44]. In relation to ecstasy, where reported effects include improved sexual performance and satisfaction [13,14], participants also reported enhanced sensuality rather than sexuality [17] and increased feelings of intimacy and emotional closeness [20,21]. Such effects may compensate for the negative effects associated with condom use such as decreased sensuality and sexual satisfaction. These effects may account for the lack of consistency of findings in relation to ecstasy observed across different studies included in our review. However, it is important to acknowledge that the pooled estimate of association between ecstasy and HIV infection was significant in case-control and longitudinal studies which provided stronger evidence than cross-sectional studies. This finding may suggest that a more robust approach to study the relationship between ecstasy and HIV infection should be explored in future studies.

Our review highlights the methodological limitations of current research. First, many studies used composite measures of drug use (e.g. any drug use) which ignore the different effects of specific drugs on sexual behaviour and ultimately on HIV transmission. Second, most studies used global measures of ATS use (that is measures unrelated to sexual encounters) with various recall periods from one month to lifetime use. Only five articles [8,51,55,58,63] reported situational or contextual drug use in which ATS were taken before or during sexual intercourse, but not during a specific event. As early as 1993, Leigh and Stall [95] recommended the use of event-specific measures of ATS use in relation to sexual encounters to enable assessment of the causal relationship between ATS use and HIV infection. Our review, conducted in 2013, was unable to find any studies which used the recommended measures. Third, a number of studies, including reviews, explored the relationship between ATS use and HIV infection [5,45,96] but not its nature or pathway; therefore, the question about causality of this relationship remains largely unanswered. Future research should take into account the methodological limitations of current studies on ATS use. Studies should adopt study designs, sampling methods and ATS use measures which would allow investigating and better understanding the temporal relationship between ATS use and HIV infection among MSM. Our analysis found that most studies were also based on opportunistic samples recruited from different source populations. Our finding of a higher pooled prevalence ratio in cross-sectional studies using samples purely recruited from clinical settings, compared to studies which relied on community-based and/or other recruitment approaches may be explained by the higher prevalence of ATS use and HIV infection among clinic patients.

Our review also identified an important gap in current research. While ATS use and HIV infections among MSM are increasing in many settings, there is little published research from LMIC. We excluded 27 articles published in languages other than English. Since 25 of them were from studies conducted in LMIC countries, it is possible that research from these countries is underrepresented in this analysis. We were not able to assess whether these studies investigated the association between ATS use and HIV infection. We found only five studies published in English language conducted in LMIC compared to 30 in high-income countries (all five studies were cross-sectional in design). As such, generalization of the relationship between ATS use and HIV infection to LMIC may not be appropriate. Further investigation is warranted in regions where ATS use is highly prevalent, such as South East Asia, and may be an important co-factor in increasing HIV transmission among MSM [97].

Our study has limitations that should be born in mind in interpreting the results. As with all meta-analyses, we were restricted to data from reports written in English [88]. Our meta-analysis cannot improve the quality of the results reported by the original studies and depends on their validity. The study diversity with respect to designs, sampling frames, populations, ATS use measures and other drug use measurement, and the heterogeneity of their results, particularly in cross-sectional and longitudinal studies, may have implications for our pooled estimates of the association between ATS use and HIV infection. We assessed heterogeneity of cross-sectional studies but unfortunately we were not able to do the same analysis for other study designs due to the small number of published articles from the longitudinal and case-control studies. They leave a potential for biased results and limit their generalizability. An inherent limitation of meta-analysis is that we could only analyze the role of ATS use in explaining the variance in HIV infections, and could not account for the possibility of various confounding factors which could also explain the association between ATS use and HIV infections (e.g. the concurrent injecting of drugs, specific sexual practices and characteristics of MSM and their networks which are the known risk factors for HIV infection). We should also acknowledge that the cross-sectional or case-control studies pooled together do not provide information about the temporal sequence between ATS use and HIV infection and, therefore, cannot attest to the causality of this relationship.

## Conclusions

The findings from our meta-analysis confirmed the significant association between meth/amphetamine use and HIV infection in all study designs, but there is lack of evidence (particularly in cross-sectional studies) regarding the role of ecstasy in HIV infection. Our review and meta-analysis also revealed important methodological limitations as to the currently used measures of drug use and their ability to establish the causal relationship between ATS use and HIV infection. Finally, our results have implications for policy and practice. Because ATS are often used in the context of high-risk unprotected sex, particularly among more adventurous MSM [11], and a significant number of HIV infections happen in these contexts [98], HIV prevention programmes targeting MSM should take into account the role of ATS use, particularly meth/amphetamines, in HIV transmission. They should also consider including interventions designed to address meth/amphetamine use in this population and adopt novel HIV prevention approaches for MSM at high risk for HIV.

## Competing interests

No declared competing interests.

## Authors' contributions

Nga Thi Thu Vu contributed significant efforts in the development and conduct of the review, performance of the statistical analysis and drafting of the manuscript. Iryna Zablotska and Lisa Maher provided oversight in the design, implementation and interpretation of findings and provided significant input into the preparation of this manuscript. All authors have seen and approved the final version of this paper.

## Supplementary Material

Amphetamine-type stimulants and HIV infection among men who have sex with men: implications on HIV research and prevention from a systematic review and meta-analysis
