# Supplementary material for: Amphetamine-type stimulants and HIV infection among men who have sex with men: implications on HIV research and prevention from a systematic review and meta-analysis
Source: J Int AIDS Soc. 2015 Feb 2;18(1):19273. doi: 10.7448/IAS.18.1.19273 (PMC4302169; doi:10.7448/IAS.18.1.19273)
Supplement: Amphetamine-type stimulants and HIV infection among men who have sex with men: implications on HIV research and prevention from a systematic review and meta-analysis [file JIAS-18-19273-s001.docx]

**Appendix 1: Quality Assessment Criteria (QAC)**

1. QAC for cross-sectional studies

|  | **Ref.number:………………………………………** | **First author, year:** |  |
| --- | --- | --- | --- |
| Q1 | Was the population from which the sample was drawn clearly stated | Background inf provided: study location, recruitment location clearly defined | 1 |
|  |  | Background inf provided: study location, recruitment location is NOT clearly defined | 0 |
| Q2 | Sampling Method | Probability sampling (simple random, systematic, stratified, cluster, multi-stage, RDS, TLS) | 1 |
|  |  | Non probability sampling (convinient, snowball, purposive, quota…) | 0 |
| Q3 | Sample represent the target population | Includion criteria of study participants clearly defined | 1 |
|  |  | Includion criteria of study participants is NOT clearly defined | 0 |
| Q4 | Response rate | Response rate >80% | 1 |
|  |  | Not mention or <80% | 0 |
| Q5 | Data collection method standardized | Identical methods of assessment and data collection used for all participants | 1 |
|  |  | Identical methods of assessment and data collection were NOT used for all participants | 0 |
| Q6 | Measure reliable | Survey instrument: tested-retested, piloted, adapted/adapted from other studies (with reference); HIV tests described (name of the test, procedure of testing) | 1 |
|  |  | Survey instrument was NOT/or MENTIONED tested-retested, piloted, adapted/adapted from other studies (with reference); HIV tests was NOT described | 0 |
| Q7 | Measure is valid | Study duration of behavioral variable was clearly defined | 1 |
|  |  | Study duration of behavioral variable was NOT clearly defined | 0 |
| Q8 | Statistical method appropriate | Yes, confident interval or SD/variance given for prevalence test | 1 |
|  |  | No, only prevalence was given | 0 |
| Q9 | Confounder management | Potential confounders not addressed in the design and analysis | 1 |
|  |  | Potential confounders addressed in the design and analysis | 0 |

1. QAC for case-control studies

|  | **Ref.number:………………………………………** | **First author, year:** |  |
| --- | --- | --- | --- |
| Q1 | Was the population from which the sample was drawn clearly stated | Background inf provided: study location, recruitment location clearly defined | 1 |
|  |  | Background inf provided: study location, recruitment location is NOT clearly defined | 0 |
| Q2 | Sampling Method | Probability sampling (simple random, systematic, stratified, cluster, multi-stage, RDS, TLS) | 1 |
|  |  | Non probability sampling (convinient, snowball, purposive, quota…) | 0 |
| Q3 | Sample represent the target population | Case and control clearly defined and from a common base represent for the target pop | 1 |
|  |  | Case and control clearly defined and NOT from a common base represent for the target pop, Control did not have the outcome at the beginning | 0 |
| Q4 | Response rate | Response rate >80% | 1 |
|  |  | Not mention or <80% | 0 |
| Q6 | Data collection method standardized | Identical methods of assessment and data collection used for all participants | 1 |
|  |  | Identical methods of assessment and data collection were NOT used for all participants | 0 |
| Q7 | Measure reliable | Survey instrument: tested-retested, piloted, adapted/adapted from other studies (with reference); HIV tests described (name of the test, procedure of testing) | 1 |
|  |  | Survey instrument was NOT/or MENTIONED tested-retested, piloted, adapted/adapted from other studies (with reference); HIV tests was NOT described | 0 |
| Q8 | Measure is valid | Study duration of behavioral variable was clearly defined | 1 |
|  |  | Study duration of behavioral variable was NOT clearly defined | 0 |
| Q9 | Statistical method appropriate | Yes, confident interval or SD/variance given for prevalence test | 1 |
|  |  | No, only prevalence was given | 0 |
| Q10 | Confounder management | Potential confounders not addressed in the design and analysis | 1 |
|  |  | Potential confounders addressed in the design and analysis | 0 |

1. QAC for longitudinal studies

|  | **Ref.number:………………………………………** | **First author, year:** |  |
| --- | --- | --- | --- |
| Q1 | Was the population from which the sample was drawn clearly stated | Background inf provided: study location, recruitment location clearly defined | 1 |
|  |  | Background inf provided: study location, recruitment location is NOT clearly defined | 0 |
| Q2 | Sampling Method | Probability sampling (simple random, systematic, stratified, cluster, multi-stage, RDS, TLS) | 1 |
|  |  | Non probability sampling (convinient, snowball, purposive, quota…) | 0 |
| Q3 | Sample represent the target population | Inclusion and exclusion criteria clearly defined, free of HIV at the beginning is clearly defined | 1 |
|  |  | Inclusion and exclusion criteria NOT clearly defined, free of HIV at the beginning is NOT clearly defined | 0 |
| Q4 | Response rate | Response rate >80% | 1 |
|  |  | Not mention or <80% | 0 |
| Q5 | Follow up rate | Follow up rate mention, charateristic of lost to follow up is NOT DIFFERENT from those of follow up | 1 |
|  |  | Follow up rate not mention, charateristic of lost to follow up is DIFFERENT from those of follow up | 0 |
| Q5 | Follow up time | Follow up time is edequate for the outcome to be occurred |  |
|  |  | Follow up time is NOT edequate for the outcome to be occurred |  |
| Q6 | Data collection method standardized | Identical methods of assessment and data collection used for all participants | 1 |
|  |  | Identical methods of assessment and data collection were NOT used for all participants | 0 |
| Q7 | Measure reliable | Survey instrument: tested-retested, piloted, adapted/adapted from other studies (with reference); HIV tests described (name of the test, procedure of testing) | 1 |
|  |  | Survey instrument was NOT/or MENTIONED tested-retested, piloted, adapted/adapted from other studies (with reference); HIV tests was NOT described | 0 |
| Q8 | Measure is valid | Study duration of behavioral variable was clearly defined | 1 |
|  |  | Study duration of behavioral variable was NOT clearly defined | 0 |
| Q9 | Statistical method appropriate | Yes, confident interval or SD/variance given for prevalence test | 1 |
|  |  | No, only prevalence was given | 0 |
| Q10 | Confounder management | Potential confounders not addressed in the design and analysis | 1 |
|  |  | Potential confounders addressed in the design and analysis | 0 |

**Appendix 2. Funnel plot and test of small sample size effect**

Figure 1. Funnel plot of total records of ATS use from selected articles

**Egger's test for small-study effects: Regress standard normal deviate of intervention effect estimate against its standard error**

**Appendix 3. Univariate Meta regression results of cross-sectional studies**

|  | Regression Coefficient (SE) | t | P value |
| --- | --- | --- | --- |
| Location of studies (developing vs. developed) | 0.72 (0.13) | -1.78 | 0.086 |
| Quality score (low vs. high) | 1.27 (0.33) | 0.93 | 0.361 |
| Sampling approach (clinic vs. other approach) | 1.66 (0.28) | 3.04 | 0.005 |
| Nitrite measure (Yes vs. No) | 0.83 (0.14) | -1.13 | 0.268 |
| ATS group (ecstasy vs. meth/amphetamine) | 0.59 (0.13) | -2.48 | 0.020 |
| Reported injecting drug use (Yes/No) | 1.02 (0.19) | 0.11 | 0.91 |

**Appendix 4. Subgroup analysis results for cross-sectional studies**

1. Subgroup analysis by location of studies

1. Subgroup analysis by quality score of studies

1. Subgroup analysis by measuring nitrite or not

1. Subgroup analysis by sampling venue

1. Subgroup analysis by ATS group

1. Subgroup analysis by reported injecting drug use

**Appendix 5: Sensitivity analysis**

1. **Sensitivity analysis for cross-sectional studies**

*Note: Quang, 2012 is Pham et al, 2012

1. Case-control studies

1. Longitudinal studies
